# How Media Exposure, Media Trust, and Media Bias Perception Influence Public Evaluation of COVID-19 Pandemic in International Metropolises

**DOI:** 10.3390/ijerph19073942

**Published:** 2022-03-25

**Authors:** Ruixia Han, Jian Xu, David Pan

**Affiliations:** 1 School of Media and Communication, Shanghai Jiao Tong University, 800 Dongchuan RD, Shanghai 200240, China; david.pan@aaagame.com; 2 Institute of Cultural Innovation and Youth Development, Shanghai Jiao Tong University, 800 Dongchuan RD, Shanghai 200240, China; 3 China Institute for Urban Governance, Shanghai Jiao Tong University, 1954 Huashan RD, Shanghai 200052, China

**Keywords:** international metropolis, pandemic evaluation, media exposure, media trust, media bias perception

## Abstract

International metropolises are key sites of outbreaks of COVID-19 cases. Global public evaluation of the pandemic in international cities is affected by many factors. This study examines how media exposure affects this evaluation and how media trust and media bias perception moderate the relationship between them. Based on an online survey of the evaluation of 13 international cities’ pandemic performances by 1171 citizens from 11 countries, this study conducted a multi-level stepwise regression analysis and discovered that: (1) different forms of media affect global citizens’ perceptions of international metropolis COVID-19 pandemic performance differently; and the role of traditional paper media, including newspapers and magazines, is of little significance in comparison to electronic media. (2) Among electronic media, TV and broadcasting have the greatest impact, followed by social media and the Internet. (3) Media trust and media bias perception affect people’s evaluations of international urban pandemics, but our survey reveals that they only function with regard to social media.

## 1. Introduction

The COVID-19 pandemic began to spread globally at the end of 2019, and densely populated cities around the world became susceptible to pandemic outbreaks. According to the number of infections announced by the World Health Organization, countries such as the United Kingdom, Germany, France, Italy, the United States, Brazil, China, India, South Africa, and Australia all have faced substantial rates of infection [1]. Each of the aforementioned nations is home to some of the world’s largest and most well-known metropolises. They hold an important status in the global political, economic, and social development network and have long been the focus in the international community. The outbreak of the pandemic has increased these cities’ global media exposure. Various types of media follow up and report the infection status, prevention measures, and treatment effects in different cities. There has been an outbreak of social media coverage of these cities due to their population diversity and the pandemic’s possible influence on them, both in breadth and depth. Citizens need to access different channels to obtain information and to facilitate their decision making during the pandemic. Under lockdown measures in different cities, regions, and countries, the media have become particularly important channels for pandemic information. In consequence, how the media influence citizens’ judgment of the pandemic has become a very important issue. Effective information dissemination can promote people to take reasonable measures to deal with COVID-19 [2], but the phenomenon of an information epidemic caused by poor information dissemination was a predicament in the early stage of epidemic management [3]. The WTO directly pointed out that the ‘infodemic’ affected people’s judgment and established an infodemic management branch. Many scholars have also proposed various countermeasures [4,5,6]. This research focuses on how individuals perceive the impact of COVID-19 on international cities and how they evaluate urban government responses to the pandemic. It examines what types of media (newspapers, television, radio, social media, etc.) are more influential in shaping public perceptions of COVID-19 in the international arena.

Another reason for widening the scope of this research to include individuals from so many nations was to incorporate the dimension of differing cultural, social, and political values. Individual reception and interpretation of information may also be affected by attitudes towards the very channel of information [7]. For example, Zhao et al., found that different trust levels of Fox News and CNN were strongly associated with whether more active protections were made [8]. The polarization of opinions on social media also affects how the public receives and evaluates online information [9]. In other words, there is not only the partisan nature of the media itself, but also the polarizing effects of information cocooning that exists in the seemingly free-flowing field of social media opinions [10]. Furthermore, hostile media effects are widely present in social life [11]. People will filter or block information presented by media with an opposing political stance or being stereotypically categorized. Regarding the global COVID-19 pandemic, extensive amounts of media coverage have taken place along all types of media channels from various countries. Our secondary aim in this research is to further explore the relationship between perceived trust in media, media bias, and individual understanding towards the global pandemic, especially in the context of countries other than their own.

To achieve this goal, we used a global network survey targeting participants from 13 representative international cities from different continents. We aim to examine how exposure to different types of media affects individual perception of the pandemic on a global scale. 

## 2. Literature Review

### 2.1. The Impact of Media Exposure on Judgment of the Pandemic

Current researches on media exposure and pandemic judgment usually have put an emphasis on media exposure’s effect on individual risk perception. For example, Huynh argued that different geographic regions, local behaviors, and customs of using social media had a positive impact on the risk perception of the COVID-19 pandemic in Vietnam [12]. Malecki et al., noticed the impact of misinformation in social media on risk tolerance or aversion [13]. A large number of studies have focused on the impact of social media use on preventative measures and behaviors taken by individuals to protect themselves from COVID-19. For example, Allington’s research contended that conspiracy theories regarding COVID-19 on social media affected people’s active behaviors, while broadcast media (television) served as a source information for the public which led to more preventative actions being taken by individuals [14]. There have also been a large number of studies conducted about the impact that media exposure has on public mental health [15,16]. Findings from Liu et al., suggested that individuals in China experienced vicarious trauma after exposure to COVID-19-related media with differing levels of trauma based on the type of media they were exposed to (official media, commercial media, social media, or overseas media) [17]. Another aspect of media exposure’s effects on public perception and attitude formation stems from the concept of the information pandemic. Many studies have found that panic regarding the pandemic is strongly tied to the rate at which misinformation is spread. For example, Cinelli et al., analyzed pandemic information on social media platforms and stated that information from both reliable and questionable sources did not present different spreading patterns, facilitating the amplification and spreading of rumors [18]. Overall, the existing body of literature strongly supports the notion that exposure to different types of media and the amount of media consumed affects individual risk perception, individual preventative and protective behaviors, and mental health. Coupled with the phenomenon of misinformation being spread on social media platforms, the impact of COVID-19 extends well beyond that of a usual health pandemic. Therefore, the importance of evaluating the different types of media and their relationship with individual attitudes towards the pandemic is particularly significant. Though a great number of studies have focused on the influence of social media, it is vital to also note the role that non-social media plays. In particular, the research of Allington and Liu et al., suggested that different types of media may have different effects on the perception of risk regarding the pandemic [14,16]. Corresponding to the development process of the media, [19,20,21] we divided the media into traditional media (represented by newspapers, magazines, television, and radio), the Internet (represented by portal sites), and social media in the Web 2.0 stage. Based on this, the following assumption was made:

**Hypothesis** **1** **(H1).** *Different media exposures have different impacts on global public judgment of the pandemic situation in different cities.*

### 2.2. The Influence of Individual Trust in the Media Regarding Pandemic Perception

Previous studies have suggested that individual trust in the media plays a significant role in shaping citizen belief and behavior. For example, Tsfati’s research stated that individuals who hold greater trust in traditional media companies were less likely to be skeptical towards presidential polling results [22]. Since Tsfati’s study was published, even more people are using the Internet and social media, leading to further studies which have re-examined the relationship between individual trust of media and behavior in the context of new media sources. A 2015 study by Turcotte et al., showed that individuals were more likely to trust news sources promoted on social media rather than traditional news outlets. This is particularly true if the news was shared on social media by a real-life friend [23]. The research of Elías and Catalan found that Spanish social media, such as WhatsApp, garnered more public trust than official media sources in COVID-19 pandemic reporting [24]. However, the increasing use of social media and the presentation of fragmented and false news on social media also lead to the public’s heightened awareness of the source. Lovari used the example of Italy to assert that the spread of misinformation on social media caused the Italian Ministry of Health to begin regulating the spread of misinformation and requiring online fact-checking documentation for publications [25]. Tsai’s research found that individuals who displayed greater trust of digital media sources and social media were less likely to hold prejudice against Asian Americans [26]. In other words, trust in the media may be directly related to people’s various attitudes and behaviors towards COVID-19. Furthermore, individual trust in the media varies significantly depending on national context. Through analyzing World Values Survey data for 44 countries, Tsfati et al., discovered that state ownership of television and the level of democracy have a significant effect on citizen trust in the media [27]. Differing sources of the same media type, such as varying television networks, may also elicit different levels of trust for each, which in turn individually impacts people’s ideas and behaviors accordingly. Using the COVID-19 pandemic as an example, Zhao et al.’s research suggested that individuals’ perceived trust in Fox News and CNN were significantly related to whether or not they adopted active preventative behaviors [8]. Although fake news can be found on different media channels, people’s stereotypes about media channels do affect their level of trust in the information published on those channels, which in turn affects their attitudes and behaviors [28]. Based on these findings, the construct of trust in the media has been shown to have a direct effect on individuals’ attitudes and behaviors, while one’s country of origin and the type of media in question also influence the relationship between the two. Therefore, we put forward the following assumption:

**Hypothesis** **2** **(H2).** *Differences in trust of different media sources will affect the global public’s judgment of the international urban pandemic.*

### 2.3. The Impact of Media Bias Perception on Judgment of the Pandemic

Compared with the previous concept of trust in the media, the concept of perceived media bias furthers our understanding of how audiences interact and are influenced by the media they come into contact with under basic cognitive and perceptual mechanisms. Media bias perception is mainly related to the party orientation and ideological stance of the media [29,30]. Extensive research conducted on hostile media effects has revealed that partisanship influences individual perceptions of objectivity and portrayal of political and social issues in media [31,32]. This cognitive stereotyping will affect an individual’s ability to process new information when it is presented by a source they deem to be foreign or counter to their own political or social identity. Although the position of the media is concealed by diversity in the context of globalization, there are still multiple interpretations in facing the COVID-19 pandemic [33], and such interpretations will affect people’s evaluations of the pandemic. The research of Ardèvol-Abreu and Gil De proved that, in the USA, public perception of media bias is closely related to the overall reduction of news media consumption [34]. Ho et al., suggested that the perception of media bias was negatively correlated with general political participation but had a direct positive correlation with issue-specific participation [35]. In other words, the perception of media bias will affect people’s media usage and individual political participation. This phenomenon has been acutely observed during political elections. As far as the COVID-19 pandemic is concerned, just as the WHO defines it as an “infodemic” [18,36], the flood of information in various media spaces has made the pandemic itself a politicized event. Reports and information surrounding the pandemic simultaneously disseminated throughout both local and international media have led to differing accounts of the same issue. They lead individuals to perceive the presence of media bias in COVID-19 reporting on the media and, in turn, effect individual perception of the pandemic itself [37]. A study by Barrios et al., examined this phenomenon and revealed that partisan bias influences how factual information about the COVID-19 pandemic is perceived [38]. Calvillo’s research showed that biases due to political ideology affect people’s perceptions of COVID-19 [33]. Even on social media, there are biases throughout the information-gathering process. Zheng et al., pointed out that social media are flooded with searches for “Chinese virus pandemonium”, suggesting that individual perception of the pandemic had a ballooning effect that influenced a larger circle of people’s behaviors and perceptions as the phrase grew more popular in searches [39]. The above research supports the proposal of the following hypothesis:

**Hypothesis** **3** **(H3).** *Media bias perception will affect the global public’s judgment of the international urban pandemic.*

### 2.4. Media Trust and Media Bias Perceptions Moderate the Influence of Media Exposure on Judgment of Pandemic Perception

Many studies have shown that individual trust in the media affects media usage. For example, Kalogeropoulos et al., studied data from 35 countries and showed that media trust as a construct affected the amount of news people consumed, and the degree of individual trust in the news was closely related to the adoption of mainstream or alternative news sources [40]. Williams’ research suggested that trust in the media and news attention are closely related [41]. In addition, the results of studies by Strömbäck [42], Turcotte [23], and Schranz [43] all have pointed to the conclusion that trust in the media is closely related to media usage and media exposure. Through the findings of previous studies suggesting that media exposure has an effect on individual perceptions of the COVID-19 pandemic in different international cities, we proposed the following hypothesis:

**Hypothesis** **4** **(H4).** *Trust in the media will moderate the influence of media exposure on the global public’s judgment of the international urban pandemic.*

In fact, correlation may exist between the perception of media bias and the influence of media exposure on the public’s judgment of the pandemic. For example, Morris pointed out that people’s perceptions of media bias will affect their exposure to media. This is visible even when the identity of the party is controlled, further facilitating the phenomenon of political polarization [44]. In a similar vein, Barnidge’s research showed that the perception of media bias is closely related to the selective contact of media [45]. An extreme case of media bias seen in individuals manifested the hostile effect between media usage and political attitudes [46]. During the COVID-19 pandemic, this phenomenon has been common. For example, AlAfnan compared articles in the American Washington Post and the Chinese People’s Daily and analyzed their respective coverages of the pandemic [47]. Mainstream media in the two countries have obvious media bias due to particular agendas. Therefore, we have reason to believe that the global public’s perception of media bias affects their judgment of the pandemic towards a given city based on information obtained through various media channels. Therefore, we proposed the following hypothesis:

**Hypothesis** **5** **(H5).** *Media bias perception will moderate the influence of media exposure on the global public’s judgment of the international urban pandemic.*

**Figure 1:**
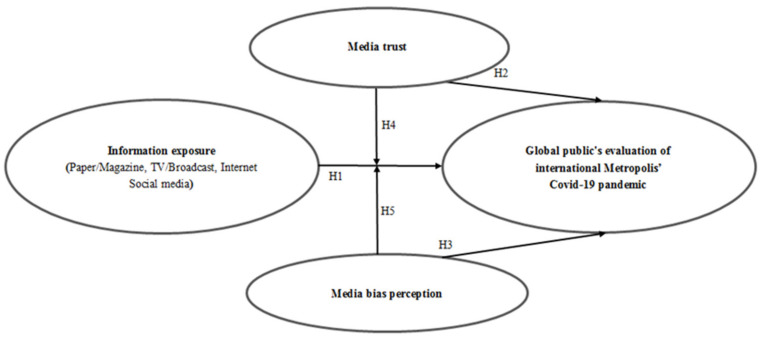
Proposed research model.

Supported by the above research assumptions, the basic model of this research was as follows (See Figure 1):

## 3. Research Design

### 3.1. Sample and Data Collection

This research relied on an online survey platform, Prolific Academic Ltd. (https://www.prolific.co/, accessed on 17–25 November 2020), to collect a total of 1242 samples from 13 countries around the world. In order to ensure the representativeness of the data, we adopted a quota sampling method for different countries and collected each country with a base of 100. Entries from Japan and Singapore were excluded because the number of collected samples was less than 50. During the data collection process, we also monitored gender ratio and the number of answers. We designed screening questions to ensure the credibility and representativeness of the collected data. A total of 1171 valid samples from 11 countries were used for data analysis. The online survey was conducted from 17 November to 25 November 2020. See Table 1 for specific demographic statistics of the sample.

### 3.2. Measurement

#### 3.2.1. Global Public Evaluation of the COVID-19 Pandemic in 13 International Cities

Global public perception of the pandemic in different international cities was the dependent variable of this study. Taking into account the global ranking of international cities (The World According to GaWC 2020), the severity of the pandemic https://covid19.who.int/ and the representation of different continents and countries, we selected 13 representative international cities, including New York, London, Paris, Tokyo, Berlin, Rome, Singapore, Sydney, Mosco, Shanghai, Johannesburg, New Delhi, and Rio de Janeiro. The specific measurement question was: On a scale of 0 to 10, how would you rate the severity of COVID-19′s impact on the following cities? Choose a value from 0–10, 10 being “Very Severe”. The overall variable value equaled the sum of the public evaluations of the pandemic situation of these cities divided by the total amount of cities.

#### 3.2.2. Media Exposure to Pandemic Information

The first influential variable examined in this study was the situation of global residents accessing COVID-19 information through various types of media. According to the development process of the media, the compatibility between each, and previous research experience [21], we divided the media into newspapers or magazines, television or broadcasts, internet sites, and social media (e.g., Facebook, Twitter, WeChat, etc.) The specific measurement question was: On a scale of 0 to 7, how much information about COVID-19 in relation to the following cities do you receive from ∗∗(media type)? 0 means “no information”, and 7 means “all my information”.

#### 3.2.3. Media Trust

Media trust measurement was mainly based on William’s combing model [41], and we examined it from three dimensions: trust of news content, trust of news reporters, and trust of news corporations. The specific measurement questions were: On a scale of 1 to 5, how much do you agree or disagree with the following statements? Items included: (1) I trust the information that I get from traditional media sources (newspapers, TV, and radio); (2) I trust the information that I find on non-social media related internet sites; (3) I trust the information that I find on social media; (4) media professionals and journalists can be trusted; and (5) media corporations can be trusted. Among them, 1–3 are informational trusts that measure different media sources, 4 measures interpersonal trust, and 5 measures institutional trust. Using the matrix list, 1 means “strongly disagree”, and 5 means “strongly agree”. The five items were integrated into the media trust index, and the coefficient of Cronbach’s alpha was 0.791.

#### 3.2.4. Media Bias Perception

According to the relevant literature review and the specific research questions of this study [41], we believe that the media biases of other countries may also superimpose the phenomenon of hostile media effects [11]. Therefore, we used two progressive questions in the measurement of this indicator: (1) I think the media are biased. (2) I think the media of other countries are more friendly and positive when reporting on their own countries. The coefficient of Cronbach’s alpha for the two questions was 0.259, indicating that they could not be combined into the same variable, so we marked them as perceived media bias 1 and perceived media bias 2. The options were still a five-degree list, with 1 for “strongly disagree” and 5 for “strongly agree”.

#### 3.2.5. Demographic Variables

The demographic variables used in this study mainly included gender, age, education, and subjective income class. The operational measurement of each indicator was as follows: gender—1 = male, 0 = female, and female as the control group; age—2020 minus the year of birth; education level—1 = high school or less, 2 = vocational school, 3 = Bachelor’s degree, and 4 = Post-graduate degree. Taking into account the differences in income levels in different countries, we adopted subjective income class indicators to measure income. The specific question was: How would you rate your income level in comparison to others in your country? The options were (1) upper, (2) upper middle, (3) middle, (4) lower middle, and (5) lower. We assigned a value of 1–5 from high to low. The average value and standard deviation of each related index are shown in Table 2.

### 3.3. Statistical Analysis

After an overall description of the evaluations of the international urban pandemic by people in different countries around the world and a simple descriptive analysis of the correlation between the influencing variables (see Table 2), we adopted the causal step regression method of Baron and Kenny [48] to create relevant assumptions for carrying out the inspection to realize, specifically through the least squares regression method found in IBM’s SPSS 19.0. We used the global public evaluation of the international metropolis COVID-19 pandemic as the dependent variable and the relevant variables as the influencing variables to gradually add into the equation process test. In the first step, we used general demographic variables and media exposure variables as independent variables in the equation to obtain model M3-1 to test H1; in the second step, we used media trust and media bias as independent variables in the equation, yielding model M3-2 to test H2 and H3; in the third step, we crossed the variables related to media trust, media bias, and media exposure and added them into the equation to obtain model M3-3 to test H4 and H5. Among them, M3-1 and M3-2 mainly examined the effects of the main variables. M3-3 examined media trust and media bias regarding the moderating effects of media exposure on public perception of the global international urban pandemic. Please refer to Section 3.2 for the internal consistency coefficient of the relevant constructive variables involved in the model.

## 4. Results

### 4.1. Descriptive Statistics

We conducted an overall analysis of the global public evaluation of the pandemic situation in different international cities. In general, the global public perceptions of the severity of the pandemic in various international cities was ranked as follows: London (M = 6.11, SD = 2.671), New York (M = 6.9, SD = 2.661), Paris (M = 5.95, SD = 2.849), Rome (M = 5.28, SD = 2.661), Rio de Janeiro (M = 4.38, SD = 3.495), Berlin (M = 4.32, SD = 2.864), New Delhi (M = 4.22, SD = 3.568), Moscow (M = 3.79, SD = 3.126), Tokyo (M = 3.46, SD = 2.696), Shanghai (M = 3.14, SD = 2.957), Sydney (M = 3.05, SD = 2.641), Johannesburg (M = 2.81, SD = 2.969), and Singapore (M = 2.79, SD = 2.65). There were significant differences between the public in different countries in the evaluation of the pandemic situation in different international cities, and the ANOVA analysis significance was all 0.00. This ranking is clearly different from the ranking of the data in November 2020 published by the World Health Organization. It can be seen that the public perception and the real pandemic situation are not completely consistent, and the influence of factors, such as the media, can be foreseen.

Based on the description of the overall pandemic situation, we conducted a basic description analysis and related analysis of the main variables involved in the study, as shown in Table 2. From this, we found that: (1) Among the demographic variables, only education affected public perception of the pandemic in different international cities. The lower the education level, the more serious the perception (r = 0.075, *p* < 0.01). (2) Among the main influencing variables involved in this study, in addition to perceived media bias 1, the categories of newspaper and magazine (r = 0.245, *p* < 0.01), TV and broadcast (r = 0.352, *p* < 0.01), the Internet (r = 0.277, *p* < 0. 01), and social media (r = 0.325, *p* < 0.01), media trust (r = 0.108, *p* < 0.01), and perceived media bias 2 (r = 0.152, *p* < 0. 01) showed significant positive correlations with perception of the international urban pandemic. (3) For other significant correlations, we found that social media exposure to COVID-19 information and media trust (r = 0.084, *p* < 0.01), and perceived media bias 2 (r = 0.059, *p* < 0.01), which reflects the effects of hostile media, showed significant correlation. More relevant results are shown in Table 2.

### 4.2. Hypothesis Testing

Table 3 presents the various relationships between media-related influencing variables and public perception of the pandemic in different international cities. Specifically, (1) M3-1 showed that, when various media-exposure-related variables were introduced, the impact of demographic variables was not significant. Among the various media types, in addition to newspaper and magazine, the other three types of TV and broadcast (β = 0.204, *p* < 0.001), the Internet (β = 0.123, *p* < 0.001), and social media (β= 0.181, *p* < 0.001) had a significant impact on global public evaluation of the international urban pandemic. In this model, TV and Broadcast had the greatest impact, and the entire model was significant (*p* < 0.001). That is to say, the impacts of various media on public perception of the international urban pandemic were different, which verified H1. It also showed that traditional media still has strong credibility in emergent public health incidents. (2) M3-2 showed that, when media trust and media bias perception variables were further introduced, it was found that, while the original significant variables of TV and broadcast (β = 0.207, *p* < 0.001), the Internet (β = 0.116, *p* < 0. 001), social media (β = 0.173, *p* < 0.01), media trust (β = 0.060, *p* < 0.05), and perceived media bias 2 (β = 0.063, *p* < 0.05) have significant influences, perceived media bias 1 had a significant edge (β = 0.056, *p* = 0.059). Overall, this verified H2 and H3. (3) M3-2 showed that, when the relevant cross-variables examining the moderating effect were introduced, the results showed that, among the relevant variables that originally played a significant role, only social media and perceived media bias 1 had significant effects, and the effects of other variables were no longer significant. Among the cross-variables, social media × media trust, social media × perceived media bias 1, and social media × perceived media bias 2 had significant effects, while the other cross-variables were not significant, which means that media trust and media bias perception fully moderate social media exposure’s influence on global public perception. This result showed the influence mechanism of social media use on judgment of the international public on the global pandemic. The specific influence direction can be shown: media trust and media bias perception 1 negatively moderated the influence of social media on judgment of the pandemic, while media bias perception 2, which reflects the hostile media phenomenon, positively moderated the influence of social media on judgment of the pandemic. This means that social media users who trust the media and believe that the media are biased have a relatively lighter estimate of the pandemic, while social media users who believe that there is a hostile media phenomenon have a heavier estimate of the pandemic. The research results partially verified H4 and H5. (4) The three regression models were significant and the adjusted R^2^ coefficient gradually increased, indicating that our research assumption of using media trust and media bias perception to predict the influence of media exposure on global public judgment and perception of the pandemic is valid.

## 5. Conclusions and Discussion

This study collected 11 countries’ public perceptions on the pandemic in 13 cities as the object and examined the impact of media exposure on them. The regression analysis results showed the following: (1) Media exposure of pandemic information is still an important factor affecting global public perception of the international urban pandemic. Contrastingly, demographic variables had no significant effect on global public perception of the pandemic. (2) Different types of media have different influences on global public perception of the international urban pandemic. Traditional paper media (newspapers and magazines) played a minimal role, while the effect of electronic media was more visible. This may be related to the impact of paper media distribution during the pandemic. Among electronic media, TV and broadcasting had the greatest influence, followed by social media and the Internet. Once again, long-standing major media organizations were still the opinion leaders of this pandemic assessment. (3) Media trust and perception of media bias significantly affected public judgment of the international urban pandemic. The overall trust in media information, practitioners, institutions, social media, and the Internet that reduced the gatekeeper effect [49] significantly affected the public perception and judgment of pandemic-related issues. The more the public trusted the media, the more serious their judgment of the pandemic. In regards to the influence of media bias perception, hostile media effects are widespread, and those who believed that media reports are internal and external demonstrated more serious judgment the pandemic. (4) The moderating role of media trust and perception of media bias was mainly active in the assessment of public perception of the international urban pandemic by social media. Access to pandemic information through social media aggravated people’s evaluations of the pandemic, but trust in the media reduced people’s perceptions of the severity of the pandemic. Similarly, the more people believed in the bias of the media, the less their judgment of the severity of the pandemic. It should be noted that, if people believed that the media reported a difference between their home country and other countries, their perception of hostile media effects was greater, and their assessment of the severity of the international urban pandemic increased.

In general, this study confirmed the influence of media exposure on global public perception of the international urban pandemic situation. It compared the effects of different influencing factors and discovered that TV and broadcast exposure causes the public’s judgment of the pandemic to be more serious. This partly responds to Allington’s research [14], which argued that traditional media with information authority makes it easier for the public to judge the severity of the pandemic and take active protective actions. At the same time, our study also responded to the current situation in which pandemic information is confusing due to the fact that, on social media, rumors cause people to panic [18]. This research showed that social media pandemic information is not as significant as TV and broadcast contact in influencing people’s judgment of the pandemic. Similar to Liu’s research [17], we distinguished the roles of different types of media in the perception and judgment of pandemic risk. The diversification and fragmentation of social media information is not as effective as traditional media (TV and broadcasting) in the face of a scientific object, such as the “pandemic”.

The research results also proved that media trust and media bias perceptions affect people’s judgment of the pandemic and moderate the impact of media exposure, especially social media exposure to pandemic information, on public perception of the international urban pandemic. This moderating role of social media showed that social media have indeed assumed the role of a secondary filter of information. In response to studies by Turcotte [23], Elías, Catalan [24], and others, media trust reduces the degree to which people judge the severity of the pandemic brought about by various information contacts on social media. That is, if people had more trust in authoritative media organizations and experts and, at the same time, had no strong resistance to information on the Internet and social media, their perceptions of the severity of the pandemic in various international cities decreased. This effect was not significant in other media types. This mode of action is still evident in the moderating effect of media bias perception on the relationship between media exposure and pandemic perception. A study by Barrios et al., confirmed that partisan bias affected people’s perceptions of factual knowledge of the COVID-19 pandemic [38]. Calvillo also explained that the public’s political ideology bias affected people’s perceptions of COVID-19 [33]. Our research further illustrates how public perception of this prejudice itself affected judgments about the pandemic. The results showed that, in terms of the impact of social media information, people’s perceptions of media bias reduced the pressure on the severity of the pandemic brought by social media information. At the same time, people’s internal awareness of the hostile media phenomena improved the judgment of the impact of social media information contact on the severity of the international urban pandemic [11,32].

Our research confirmed how media exposure, media trust, and media bias perception influence global public judgment of the international urban pandemic. It demonstrated the progress in the mechanism of this effect. It analyzed the mediating role of media trust and media bias perception in the relationship between social media exposure to pandemic information and public perception of the pandemic. This discovery has practical significance for promoting the governance of the global pandemic under the situation that the pandemic is still continuing. That is, although the information chaos of social media has caused the pandemic to show the characteristics of an information pandemic, in fact, traditional media trust and public media bias perception is still regulating the generation of various types of information on people’s perceptions of the pandemic. Different countries are further strengthening the authority and factuality of mainstream media information and attach importance to the voices of relevant institutions and experts on social media, which can effectively improve the factual judgment of the public’s perception of the pandemic in different countries, help them make plans, and objectively promote global prevention.

It should be noted that the research in this article focused on revealing how media-related factors affect public perception of the international urban pandemic. Therefore, factors other than demographic variables were not covered in this research. In fact, emotions, cultural values, and other factors that contribute to the generation of perceptions are suitable for testing and comparison using the same model, forming a comprehensive factor model for pandemic evaluation. In terms of sample composition, due to the online survey, the overall sample population was relatively young. In addition, this research focused on the exploration of macro-mechanisms, so there was no detailed comparative analysis of the factors affecting the evaluation of the pandemic situation of the people from different countries in various international cities. We hope to further address the aforementioned points in subsequent studies. We also look forward to the emergence of studies that more closely measure the relationship between local media exposure and pandemic judgment in international cities.

## Figures and Tables

**Table 1:** Distribution of sample sociodemographics (n = 1171).

Variables	Categories	Frequency	Percentage (%)
Gender	Male	624	51.3
	Female	547	45.0
Education	Junior high school and below	301	24.8
	High school	83	6.8
	College/University	453	37.3
	Master and above	334	27.5
Subjective income class	Upper	129	10.6
	Upper middle	277	22.8
	Middle	529	43.5
	Lower middle	216	17.8
	Lower	42	7.5
Nationality	Australia	106	8.7
	Brazil	110	9.0
	China	102	8.4
	France	112	9.2
	Germany	108	8.9
	India	111	9.1
	Italy	111	9.1
	Russia	85	7.0
	South Africa	124	10.2
	UK	102	8.4
	US	100	8.2
Age	Mean	29.9	

**Table 2:** Means, standard deviations, and intercorrelations of measurements.

	Gender	Age	Education	Income	Paper/ Magazine	TV/ Broadcast	Internet	Social Media	Media Trust	Perceived Media Bias 1	Perceived Media Bias 2	Public Evaluation
Gender												
Age	−0.025											
Education	−0.033	0.190 **										
Income	0.034	0.067 *	0.245 **									
Paper/Magazine	−0.015	0.081 **	0.107 **	0.148 **								
TV/Broadcast	0.059 *	0.127 **	0.051	0.092 **	0.527 **							
Internet	0.134 *	0.106 **	0.116 **	0.024	0.336 **	0.380 **						
Social Media	0.018 **	−0.073 *	0.024	0.047	0.341 **	0.466 **	0.304 **					
Media Trust	−0.007	0.030	0.019	0.095 **	0.173 **	0.141 **	0.083 **	0.084 **				
Perceived Media Bias 1	−0.005	−0.056	0.030	−0.026	−0.159 **	−0.162 **	−0.037	−0.011	−0.321 **			
Perceived Media Bias 2	0.025	−0.054	−0.038	0.012	0.008	0.018	0.055	0.059 *	0.104 **	0.149 **		
Public Evaluation	−0.050	0.031	0.075 *	0.043	0.24 5 **	0.352 **	0.277 **	0.325 **	0.108 **	0.008	0.102 **	
Mean	0.47	29.91	2.70	2.76	1.9289	2.3489	2.3943	2.9607	2.5959	3.76	3.16	4.4394
SD	0.499	10.635	1.138	0.935	1.83348	1.79415	1.92431	2.38636	0.75591	1.096	1.146	2.09252

Notes: * *p* < 0. 05, ** *p* < 0. 01.

**Table 3:** Multiple regression analysis for global public evaluation of the COVID-19 pandemic of different metropolises.

	Variables	M3-1			M3-2			M3-3		
		B	SE	β	B	SE	β	B	SE	β
	Gender	0.082	0.116	0.020	0.081	0.116	0.019	0.078	0.116	0.019
	Age	−0.002	0.006	−0.010	0.000	0.006	−0.001	0.001	0.006	0.004
	Education	−0.007	0.064	−0.003	−0.020	0.064	−0.009	−0.025	0.064	−0.011 *
	Income	0.104	0.053	0.057	0.101	0.053	0.055	0.115	0.053	0.062
*Media exposure to COVID-19*	Paper/Magazine	0.029	0.039	0.025	0.030	0.039	0.027	0.290	0.244	0.254
	TV/Broadcast	0.238	0.042	0.204 ***	0.242	0.042	0.207 ***	−0.053	0.267	−0.046
	Internet	0.135	0.034	0.123 ***	0.127	0.034	0.116 ***	−0.047	0.214	−0.043
	Social media	0.159	0.028	0.181 ***	0.152	0.028	0.173 ***	0.553	0.175	0.631 **
*Moderated variables*	Media trust				0.165	0.082	0.060 *	0.213	0.159	0.077
	Perceived media bias 1				0.108	0.057	0.056 *	0.276	0.113	0.145 *
	Perceived media bias 2				0.115	0.051	0.063 *	0.110	0.104	0.060
*Media exposure* × *Media trust*	Paper/Magazine × Media trust							−0.019	0.054	−0.051
	TV/Broadcast × Media trust							0.089	0.061	0.234
	Internet × Media trust							0.051	0.048	0.137
	Social media × Media trust							−0.108	0.038	−0.362 **
*Media exposure* × *Media bias 1*	Paper/Magazine × Perceived media bias 1							−0.026	0.037	−0.089
	TV/Broadcast × Perceived media bias 1							0.034	0.041	0.113
	Internet × Perceived media bias 1							0.012	0.032	0.047
	Social media × Perceived media bias 1							−0.073	0.028	−0.347 *
*Media exposure* × *Media bias 2*	Paper/Magazine × Perceived media bias 2							−0.032	0.032	−0.102
	TV/Broadcast × Perceived media bias 2							−0.023	0.035	−0.074
	Internet × Perceived media bias 2							−0.005	0.029	−0.015
	Social media × Perceived media bias 2							0.048	0.023	0.207 *
	F	29.335 ***			22.785 ***			11.834 ***		
	Adjusted R²	0.169			0.177			0.183		
	ΔR²	0.175			0.185			0.200		

Notes: * *p* < 0.05, ** *p* < 0.01, *** *p* < 0.001.

## Data Availability

The raw data supporting the conclusions of this article will be made available by the authors without undue reservation.
